# Proteomic Analysis of Low-Temperature Stress Response in Maize (*Zea mays* L.) at the Seedling Stage

**DOI:** 10.3390/cimb47090784

**Published:** 2025-09-22

**Authors:** Tao Yu, Jianguo Zhang, Xuena Ma, Shiliang Cao, Wenyue Li, Gengbin Yang

**Affiliations:** 1 Maize Research Institute of Heilongjiang Academy of Agricultural Sciences, Harbin 150086, Chinakshmaize@163.com (G.Y.); 2 Key Laboratory of Biology and Genetics Improvement of Maize in Northern Northeast Region, Ministry of Agriculture and Rural Affairs, Harbin 150086, China; 3 Key Laboratory of Germplasm Resources Creation and Utilization of Maize, Harbin 150086, China; 4 Sanya Institute of China Agricultural University, Sanya 572024, China

**Keywords:** DEPs, three-leaf stage, molecular mechanisms, WGCNA

## Abstract

Low temperature severely restricts maize seedling establishment and yield in northern China, but the proteomic basis of low-temperature tolerance in maize remains unclear. This study used TMT-labeled quantitative proteomics combined with data-independent acquisition (DIA) and liquid chromatography–tandem mass spectrometry (LC-MS/MS) to analyze dynamic proteome changes in two maize inbred lines (low-temperature-tolerant B144 and low-temperature-sensitive Q319) at the three-leaf stage under 5 °C treatment. A total of 4367 non-redundant proteins were identified. For differentially expressed proteins (DEPs, fold change >2.0 or <0.5, ANOVA-adjusted *p* < 0.05, false discovery rate [FDR] < 0.05), B144 showed exclusive upregulation under stress (6 DEPs at 24 h; 16 DEPs at 48 h), while Q319 exhibited mixed regulation (9 DEPs at 24 h: 6 upregulated, 3 downregulated; 21 DEPs at 48 h: 19 upregulated, 2 downregulated). Functional annotation indicated that ribosomal proteins, oxidoreductases, glycerol-3-phosphate permease, and actin were significantly upregulated in both lines. Pathway enrichment analysis revealed associations with carbohydrate metabolism, amino acid biosynthesis, and secondary metabolite synthesis. Weighted gene co-expression network analysis (WGCNA) identified genotype-specific expression patterns: B144 showed differential expression of proteins related to acetyl-CoA synthetase and fatty acid β-oxidation at 24 h and of proteins related to D-3-phosphoglycerate dehydrogenase at 48 h; Q319 showed differential expression of proteasome-related proteins at 24 h and of proteins related to elongation factor 1α (EF-1α) at 48 h. Venn analysis found no shared DEPs between the two lines at 24 h but four overlapping DEPs at 48 h. These results clarify proteomic differences underlying low-temperature tolerance divergence between maize genotypes and provide candidate targets for molecular breeding of low-temperature-tolerant maize.

## 1. Introduction

Low temperature is a critical environmental factor that disrupts normal plant growth and development [1]. Low-temperature stress damages the structural integrity of the cellular membrane system, leading to dysregulation of cellular material exchange, protein degradation, membrane injury, and enzyme inactivation [2,3,4]. Additionally, low temperature reduces photosynthetic rates, inhibits chlorophyll and chloroplast formation, and decreases organic compound accumulation, thereby impairing plant growth. The severity of these effects escalates with lower temperatures and prolonged exposure, potentially causing plant mortality under extreme conditions [5,6]. With the increasing frequency of global climatic anomalies, the occurrence and geographic impact of low-temperature stress are expanding [7]. Under such stress, plants undergo significant alterations in the expression profiles and abundance of genes and proteins [8]. These changes enable plants to activate defense mechanisms to counteract adverse environmental conditions. As direct products of gene expression, proteins regulate most physiological functions in plants [9]. Proteomic approaches, including qualitative and quantitative analyses of differentially expressed proteins under low-temperature stress, provide critical insights into stress-responsive protein dynamics, adaptive regulatory mechanisms, and potential target identification. This facilitates a deeper understanding of plant physiological and metabolic adaptations at the protein level under abiotic stress.

The proteome represents the entire set of proteins expressed by an organism, tissue, or cell under specific conditions [10]. Introduced by Marc Wilkins in 1994 [11], proteomics focuses on exploring the composition and dynamic changes of proteins within cells, tissues, or organisms. This technology has been extensively utilized in researching plant responses to low-temperature stress [12,13,14,15]. Xing et al. conducted proteomic and phosphoproteomic analyses on cold-stressed maize seedlings, identifying 660 proteins with abundance changes and 620 proteins with site-specific phosphorylation alterations in response to low temperature. Functional enrichment analysis indicated that early cold responses in maize involve the light reactions of photosynthesis, spliceosome-related functions, endocytosis, and defense mechanisms [16]. Wang et al. applied tandem mass tag (TMT)-based quantitative proteomics to wild rice DC907. They found that the number of DEPs under cold stress increased over time and that 366 unique proteins were associated with ATP synthesis, photosystems, reactive oxygen species (ROS) regulation, stress responses, and cellular integrity [17]. Zhang et al. reported that in cold-stressed cotton seedlings, proteins related to the MAPK and calcium signaling pathways showed enhanced activity. Meanwhile, the metabolism of carbohydrates, lipids, nucleotides, and amino acids was upregulated, and antioxidant enzymes were activated to reduce oxidative damage [18]. Li et al. used 2-DE and LC-ESI-MS/MS to identify 52 DEPs in the potato cultivar “Zhengshu No. 6”; these DEPs were mainly involved in defense responses, energy metabolism, and photosynthesis [19]. A study on wheat seedlings exposed to 4 °C for 48 h identified 45 DEPs. Photosystem-related proteins were significantly downregulated, while antioxidant enzymes and chaperones were upregulated, suggesting that cold stress suppresses photosynthesis and activates antioxidant defenses in wheat [20]. Comparative proteomics between cold-tolerant and cold-sensitive varieties has been instrumental in identifying biomarkers for molecular breeding. For example, Tang et al. used iTRAQ technology to analyze rice roots under 5 °C stress. They identified 167 and 261 DEPs in Dongxiang wild rice and Zhongjiazao 17, respectively. Shared pathways included carbon metabolism and secondary metabolite biosynthesis, highlighting energy reinforcement as a crucial cold tolerance strategy [21]. Long et al. identified 416 DEPs in sugar beet chloroplasts under low temperature. These DEPs were involved in photosynthetic protein transport (BvLTD and BvTOC100), starch granule formation (BvPU1, BvISA3, BvGWD3), and ROS scavenging (BvCu/Zn-SOD, BvCAT, BvPrx, BvTrx), which are important for early cold adaptation [22].

Maize (*Zea mays* L.) is a globally crucial crop, playing a significant role in China’s agriculture. As a thermophilic plant, maize thrives within a relatively narrow temperature range, with an optimal growth temperature of 25–28 °C and a minimum tolerable temperature of 5–18 °C [23]. Maize exhibits high sensitivity to low temperatures. A mere 0.7 °C decrease in temperature during its growth and development stage can delay the maturity period by 7 days, leading to an 8% reduction in yield. Moreover, a further 1 °C drop in temperature may extend the total growth period of maize by 10 days and cause a yield reduction of over 10%. Chilling injury symptoms become apparent when the temperature drops to ≤10 °C [24]. This temperature decrease also shortens the grain-filling period, which not only results in empty stalks, ear baldness, and insufficient grain formation but also increases the height, leaf age, and dry matter content of maize seedlings. In recent years, frequent spring chilling events in northern China’s maize-growing regions have severely hampered seed germination and seedling growth, resulting in yield losses ranging from 20% to 30% [24]. Advances in multi-omics technologies (genomics, transcriptomics, metabolomics, proteomics) have greatly enhanced the mechanistic understanding of plant abiotic stress responses [25]. However, current proteomic studies on maize in China predominantly focus on salt stress [26] and biotic stress [27], with limited exploration of low-temperature stress. In a related study, we screened a comprehensive collection of maize inbred lines and identified two distinct lines, B144 and Q319, which exhibit insensitivity and sensitivity to cold stress, respectively [24]. This research delves into the proteomes of B144 and Q319 under low-temperature stress conditions. By comparing treated and control samples, we aim to identify DEPs and uncover the metabolic pathways associated with low-temperature tolerance. The results of this study lay a solid foundation for the screening of low-temperature-resistant germplasm, the development of low-temperature-tolerant maize varieties through breeding programs, and the efficient exploitation of genetic resources in maize.

## 2. Materials and Methods

### 2.1. Plant Materials and Stress Treatments

The maize inbred lines B144 (B; highly low-temperature tolerant) and Q319 (Q; low-temperature sensitive) were preserved in the germplasm repository of the Maize Research Institute, Heilongjiang Academy of Agricultural Sciences [24]. Uniform, plump seeds were sown in 24-cell plastic germination trays (37 cm × 24 cm × 8 cm) filled with vermiculite and cultivated in a climate-controlled growth chamber (day/night temperature: 25 °C; photoperiod: 16 h/8 h; relative humidity: 70%). At the three-leaf stage, seedlings were subjected to low-temperature treatments (5 °C) for 24 h (B24/Q24) and 48 h (B48/Q48), with control groups (BCK/QCK) maintained at 25 °C. Leaves from treated and control plants, with three biological replicates per group, were flash-frozen in liquid nitrogen and stored at −80 °C for proteomic analysis.

### 2.2. Protein Extraction and Quality Control

Protein extraction protocol: approximately 0.15 g of leaf tissue was homogenized in a 1.5 mL grinding tube with 700 μL of 4% SDS buffer (two grinding cycles). Samples were heated at 90 °C for 10 min, followed by two additional grinding cycles. After centrifugation (15,000× *g*, 10 min), supernatants were mixed with four volumes of pre-chilled acetone for overnight precipitation. Pellets were dissolved in 400 μL of 4% sodium dodecyl sulfate (SDS) buffer.

Quality control: protein concentrations were quantified using the bicinchoninic acid (BCA) assay, with a standard curve derived from the optical density (OD) measurements of samples at 562 nm. Protein samples (35 μg) were mixed with 4× loading buffer (4:1 *v*/*v*), heated (10 min, 100 °C), centrifuged (14,000× *g*, 10 min), and separated on 12% gels (200 V, 30 min). Gels were stained with Coomassie brilliant blue for visualization.

### 2.3. Filter-Aided Sample Preparation (FASP)

Proteins (100 μg) were reduced with 10 mM of DTT (55 °C, 30 min) and alkylated with 50 mM of iodoacetamide (room temperature, 30 min, dark). Excess reagent was quenched with 2.5 μL of DTT. Precipitated proteins (four volumes of acetone, −20 °C, 4 h) were washed twice with cold acetone, air-dried, and digested with trypsin (1:50–1:10 *w*/*w*) in 50 mM of NH_4_HCO_3_ (37 °C, overnight). Peptides were acidified with 1% TFA, desalted, and lyophilized.

### 2.4. High-pH Reverse-Phase Peptide Fractionation

Lyophilized peptides (300 μg) were fractionated using high-pH reverse-phase chromatography: columns were conditioned with methanol and 80% acetonitrile. Peptides were dissolved in 200 μL of pH 10 ammonia buffer, loaded, and eluted with a stepwise acetonitrile gradient (6%, 9%, 12%, 15%, 18%, 21%, 25%, 30%, 35%, 50%). Eluted fractions were pooled into three groups and lyophilized.

### 2.5. DDA Library Construction and DIA Quantification

Peptides were reconstituted in mobile phase A (0.1% formic acid, 2% acetonitrile), separated using an UltiMate 3000 UHPLC system (Thermo Fisher Scientific, Waltham, MA, USA), and then ionized via a nanoESI source (TriVersa NanoMate, Ithaca, NY, USA); the UHPLC separation employed an Acclaim PepMap RSLC C18 column (2.1 mm × 150 mm, 2 μm, Thermo Fisher Scientific) with a gradient elution program: 2% mobile phase B (0.1% formic acid, 98% acetonitrile) from 0 to 2 min, a linear increase from 2% to 35% mobile phase B from 2 to 40 min, a further increase from 35% to 95% mobile phase B from 40 to 42 min, maintenance of 95% mobile phase B from 42 to 45 min, and reversion to 2% mobile phase B from 45.1 to 50 min, along with a flow rate of 0.3 mL/min, column temperature of 40 °C, and injection volume of 5 μL. Mass spectrometry analysis was conducted on a Q-Exactive HF system (Thermo Fisher Scientific) using data-dependent acquisition (DDA) mode for library generation—where full scans were performed over a range of 350–1800 *m*/*z* at a resolution of 60,000, the top 20 precursor ions were fragmented via higher-energy collisional dissociation (HCD) with 30% collision energy, and MS/MS scans were acquired at a resolution of 15,000—and data-independent acquisition (DIA) mode for quantification—where full scans were also conducted over 350–1800 *m*/*z* at 60,000 resolution, 60 variable DIA windows (25 *m*/*z* width) were set, and MS/MS scans were obtained at 30,000 resolution with 28% collision energy. The nanoESI ion source was operated at a spray voltage of 2.0 kV and an ion transfer tube temperature of 320 °C.

### 2.6. Quantitative Analysis and Data Quality Assessment

In the realm of proteomic research, ensuring data integrity and reliability is crucial. For protein identification, data-dependent acquisition (DDA) raw mass spectrometry data were processed by Proteome Discoverer 2.1.0182 (Thermo Fisher Scientific) to determine the presence of target proteins. Regarding peptide quantification, data-independent acquisition (DIA) data were analyzed using Spectronaut software (Biognosys AG, Schlieren, Switzerland), which extracted peptide signals from DIA raw data for relative quantification, thereby supporting the quantification of corresponding proteins. Data quality was evaluated through three key metrics. Intensity consistency was examined by comparing the signal intensity of target peptides/proteins across replicates, as stable intensity indicated a reliable detection system. Mass accuracy was verified by comparing the measured and theoretical mass-to-charge ratio (*m*/*z*) of peptides, ensuring that deviations were within an acceptable range for accurate peptide/protein identification. Sample correlation was assessed by calculating Pearson correlation coefficients between samples, especially replicates, to ensure the reproducibility of protein expression profiles. To further validate protein identification results, three core statistical indicators were analyzed. Unique peptide counts per protein served as an indicator of identification confidence, with more unique peptides corresponding to a protein signifying a more reliable result. The distribution of protein molecular weights (in kDa) was analyzed to understand the diversity of protein sizes in samples, providing a reference for the coverage of proteins with different molecular weights. Protein sequence coverage, defined as the percentage of a protein’s amino acid sequence covered by identified peptides, reflected the comprehensiveness of protein detection, helping to confirm the integrity of identification. These integrated steps in data processing, quality evaluation, and result validation are fundamental to obtaining accurate and trustworthy proteomic data.

### 2.7. Weighted Gene Co-Expression Network Analysis (WGCNA)

Co-expression networks were constructed using the R package *WGCNA* (V1.478). A soft threshold power was selected to ensure scale-free topology. Modules were identified via dynamic tree cutting (minimum module size: 30 genes; merge threshold: 0.25). Module eigengenes (MEs) were correlated with target traits (Pearson correlation, *p* < 0.05). Functional enrichment (GO and KEGG) was performed using DAVID (*FDR* < 0.05). Hub genes were visualized in Cytoscape 3.9.1.

### 2.8. Data Processing and Bioinformatics

Statistical analyses were conducted using SAS 9.0, with results expressed as means ± SD (three replicates). DEPs were annotated via GO (Gene Ontology; http://geneontology.org; URL accessed on 15 May 2024) and KEGG (Kyoto Encyclopedia of Genes and Genomes; https://www.kegg.jp; URL accessed on 20 May 2024). Protein–protein interaction networks were analyzed using STRING.

### 2.9. Hub Gene Validation by qPCR

To validate the expression patterns of hub genes identified from the weighted gene co-expression network analysis (WGCNA), four hub genes derived, respectively, from the brown module, blue module, pink module, and turquoise module were selected for qPCR analysis. Primers for these target genes were designed using Beacon Designer software (version 7.8; http://www.premierbiosoft.com/molecular_beacons/; accessed on 8 November 2023) (Table 1), with primer specificity verified via BLAST (V2.14.0) against the maize reference genome (B73 RefGen_v4) to ensure no off-target binding. The maize *Actin1* gene (GenBank Accession Number: GRMZM2G126010) was used as the internal control to normalize variations in RNA extraction efficiency and reverse transcription (RT) reactions.

Total RNA from maize seedling samples (three biological replicates per treatment group, with two technical replicates per biological replicate) was first reverse-transcribed into cDNA using the PrimeScript™ RT Reagent Kit with gDNA Eraser (TaKaRa, Kusatsu City, Japan) to eliminate genomic DNA contamination. qPCR was then performed on an ABI7500 Real-Time System (Applied Biosystems, Waltham, MA, USA) following the manufacturer’s instructions for the SYBR^®^ Green Real-time PCR Master Mix (TOYOBO, Osaka, Japan). The 20 μL reaction system consisted of 10 μL of SYBR^®^ Green Master Mix, 0.4 μL each of forward and reverse primers (10 μM), 2 μL of cDNA template (diluted 1:5 with nuclease-free water), and 7.2 μL of nuclease-free water.

The thermal cycling conditions were set as follows: an initial denaturation step at 95 °C for 30 s to activate the DNA polymerase, followed by 40 cycles of 95 °C for 5 s (denaturation), 60 °C for 30 s (annealing), and 72 °C for 30 s (extension). After the cycling stage, a melting curve analysis (from 60 °C to 95 °C, with a temperature increment of 0.5 °C every 10 s) was conducted to confirm the specificity of the PCR products (single melting peak indicates no non-specific amplification or primer dimers).

The relative expression levels of the target genes were calculated using the 2^−∆∆CT^ method, where ∆CT = CT (target gene) − CT (*Actin1*), and ∆∆CT = ∆CT (treatment group) − ∆CT (control group). Statistical analysis of variance (ANOVA) and significance testing (*p* < 0.05) were performed using SPSS Statistics v23.0 software (IBM, New York, NY, USA) to determine differences in gene expression between groups, with results presented as the mean ± standard error (SE) of three biological replicates.

**Table 1:** Primer sequence of hub genes for qPCR.

Gene	Forward Primer	Reversed Primer
*Zm00001d016659*	CGGATCTCGCCGCCTCTCCC	GCTCGGCGCCGGCGAAGAT
*LOC100276453*	GCCGCTAGGGTTCCGGCGAG	CAGCAGCAGTCACACCCGGG
*LOC103645840*	GCCGTCCCCGACCACTACCG	GCTCGCCAACGTCGACTGCTC
*LOC103633583*	GAAAACCTATAGCTTGCAGCG	GCTCGCCAACGTCGACTGCTC

## 3. Results

### 3.1. Statistical Overview of Protein Identification

**Figure 1:**
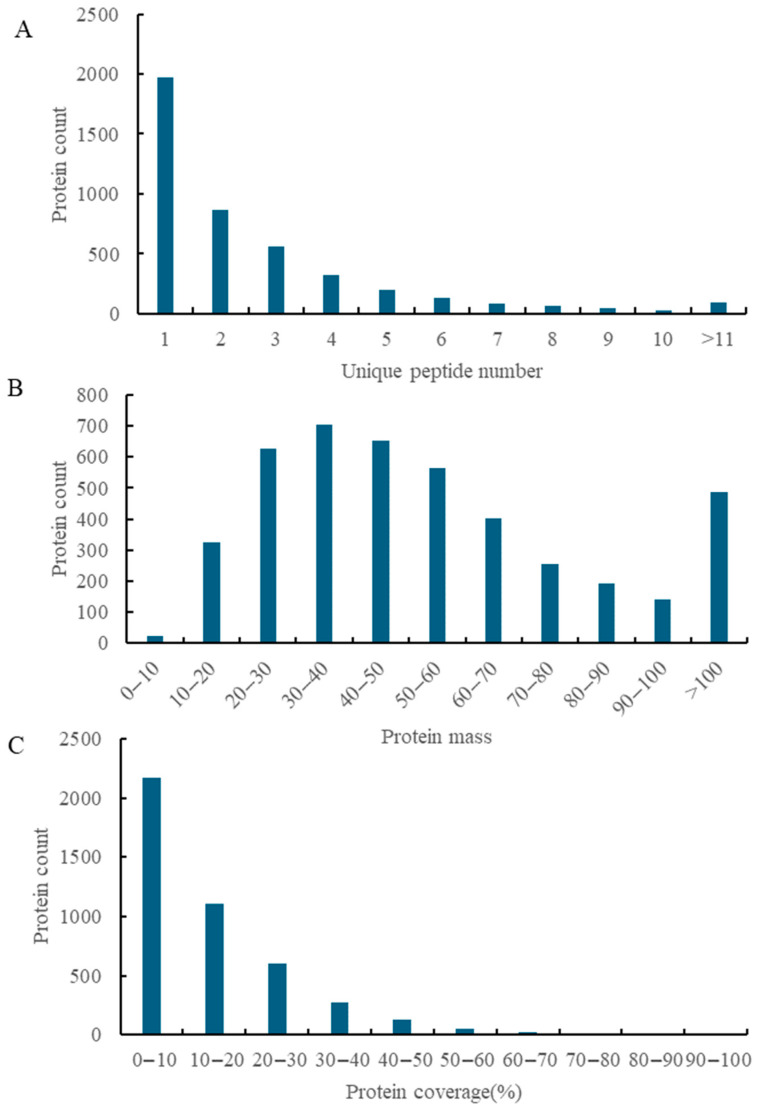
Basic statistics of protein identification. Note: (**A**) unique peptide counts per protein: X-axis = peptide count ranges (e.g., 1, 2, 3–5), Y-axis = protein number; more peptides means higher identification confidence. (**B**) Protein molecular weights: X-axis = kDa ranges (e.g., “10–20” = 10–20 kDa), Y-axis = protein number; shows diverse protein sizes. (**C**) Protein sequence coverage: X-axis = coverage percentages (e.g., 0–10%), Y-axis = protein number; higher coverage means more thorough detection.

A total of 5041 peptides corresponding to 4637 proteins were identified across 18 maize leaf samples. As shown in Figure 1A, the peptide distribution was as follows: a total of 1970 proteins (42.5%) were identified with 1 peptide, 1747 (37.7%) with 2–4 peptides, 420 (9.1%) with 5–7 peptides, 139 (3.0%) with 8–10 peptides, and 90 (1.9%) with ≥11 peptides. Figure 1B shows the protein molecular weight (MW) distribution, which is crucial for assessing identification completeness. The majority of proteins (53.33%) fell within the 10–50 kDa range, followed by 51–100 kDa (35.52%) and >100 kDa (11.15%), indicating a broad MW spectrum typical of plant proteomes. Protein sequence coverage, reflecting the proportion of a protein’s amino acid sequence covered by detected peptides, is presented in Figure 1C. The majority of proteins (49.71%, *n* = 2171) had 0–10% coverage, while 10–20% coverage accounted for 25.26% (*n* = 1103). Coverage exceeding 60% was rare (0.62%, *n* = 29), suggesting challenges in detecting low-abundance or post-translationally modified proteins.

Overall, these data comprehensively characterized the identified proteins in this study. The peptide distribution demonstrated that a large proportion of proteins were identified with a relatively small number of peptides, which might be related to the complexity of the proteome and the limitations of the detection method. The broad MW spectrum indicated that the proteomic analysis covered proteins of different sizes, suggesting a comprehensive sampling of the maize leaf proteome. The low sequence coverage for most proteins, especially the rarity of high-coverage proteins, highlighted the technical difficulties in fully characterizing the proteome, particularly in detecting low-abundance and modified proteins. However, the consistency between peptide counts, MW distribution, and expected technical performance validated the depth and reliability of the proteomic dataset, providing a solid foundation for subsequent analyses.

### 3.2. Data Quality Control

**Figure 2:**
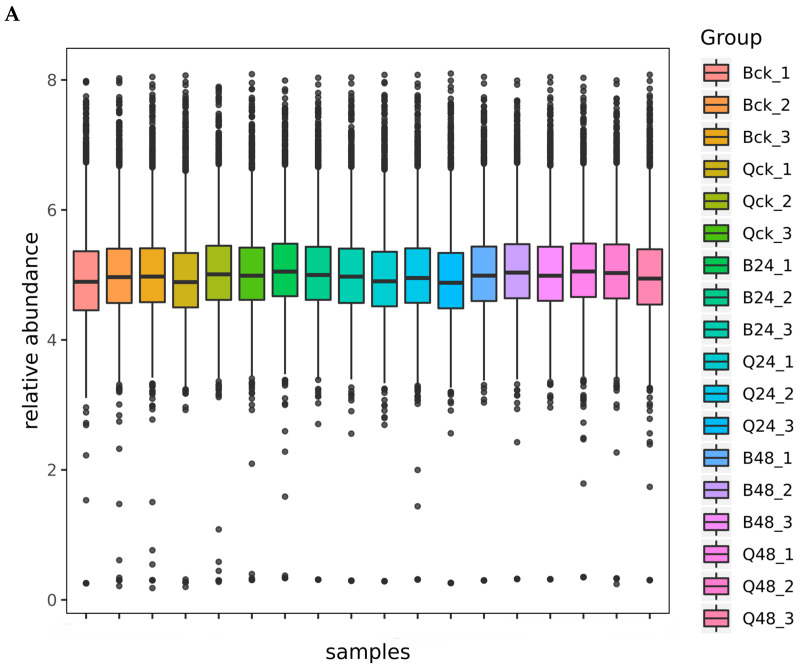

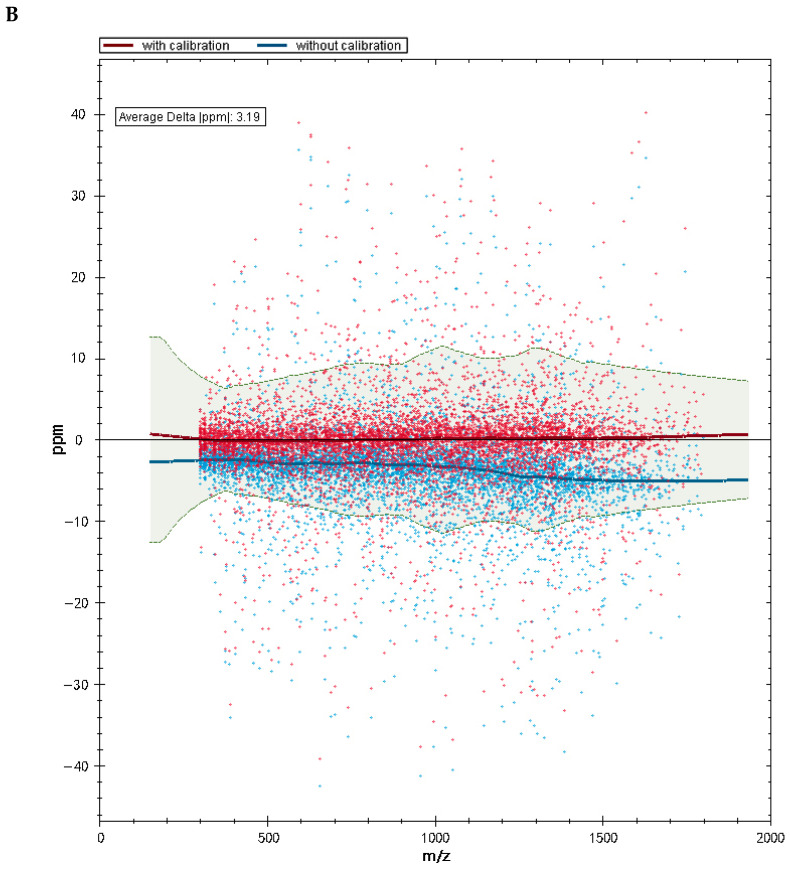
DIA data quality assessment. Note: (**A**) box plots of protein relative abundance distribution; (**B**) histogram of mass accuracy deviations (ppm) for secondary ions (y-axis) across *m*/*z* ranges (x-axis).

As shown in Figure 2A, the relative abundance distribution patterns of proteins across all 18 samples exhibited high similarity, indicating exceptional consistency in sample preparation procedures and instrument stability, which ensured the accuracy and reliability of experimental results. Figure 2B displays the histogram of mass accuracy deviations for secondary fragment ions of all quantified peptides. The narrow distribution of deviations (<5 ppm) across the entire dataset further confirms the robust instrument performance during the detection process, guaranteeing the precision of both protein identification and quantification. These quality control metrics validate the technical reproducibility of the proteomic workflow, minimizing batch effects and supporting the biological relevance of downstream analyses.

### 3.3. Hierarchical Clustering Analysis of DEPs

**Figure 3:**
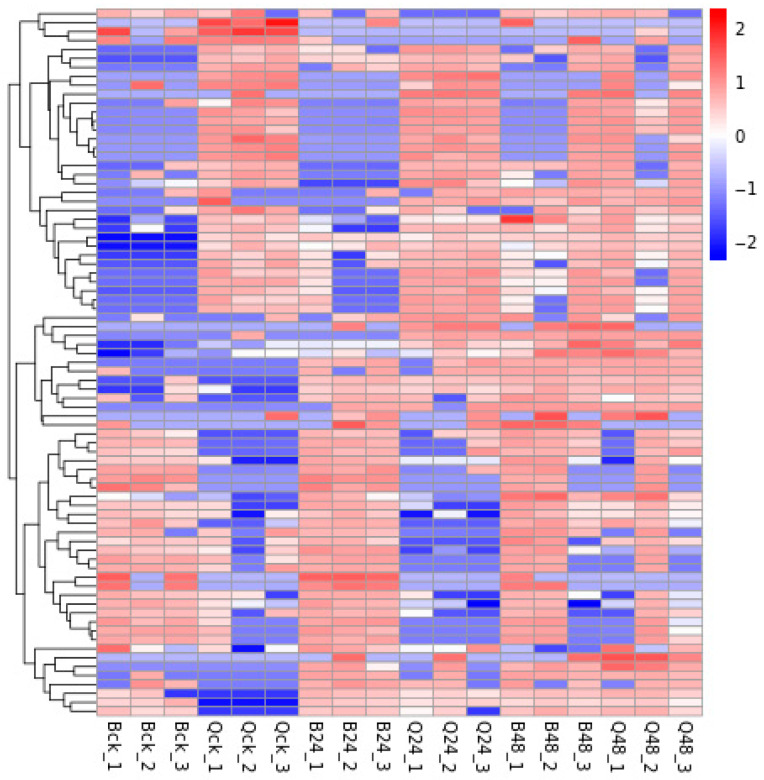
Heatmap of DEP clustering. Note: rows represent the protein clusters; columns represent sample clusters; color intensity reflects expression levels. y-axis represents individual differentially expressed proteins (DEPs) identified in the study.

To visualize the similarities and differences in DEPs in maize leaves under varying durations of low-temperature stress, hierarchical clustering analysis was performed on all identified DEPs. Red and blue colors in the heatmap (Figure 3) represent upregulated and downregulated proteins, respectively. As shown in Figure 3, distinct expression patterns were observed between low-temperature-stressed and control groups. Notably, high similarity among the three biological replicates within each treatment group underscores the reproducibility of the experimental design and validates the robustness of the DEP screening criteria. These results demonstrate that the identified DEPs reliably reflect low-temperature-stress-induced molecular adjustments, with coordinated upregulation of stress-responsive proteins (e.g., antioxidant enzymes, chaperones) and downregulation of growth-related proteins (e.g., photosynthetic machinery), aligning with the physiological demands of low-temperature acclimation.

### 3.4. Identification of DEPs Under Low-Temperature Stress

**Figure 4:**
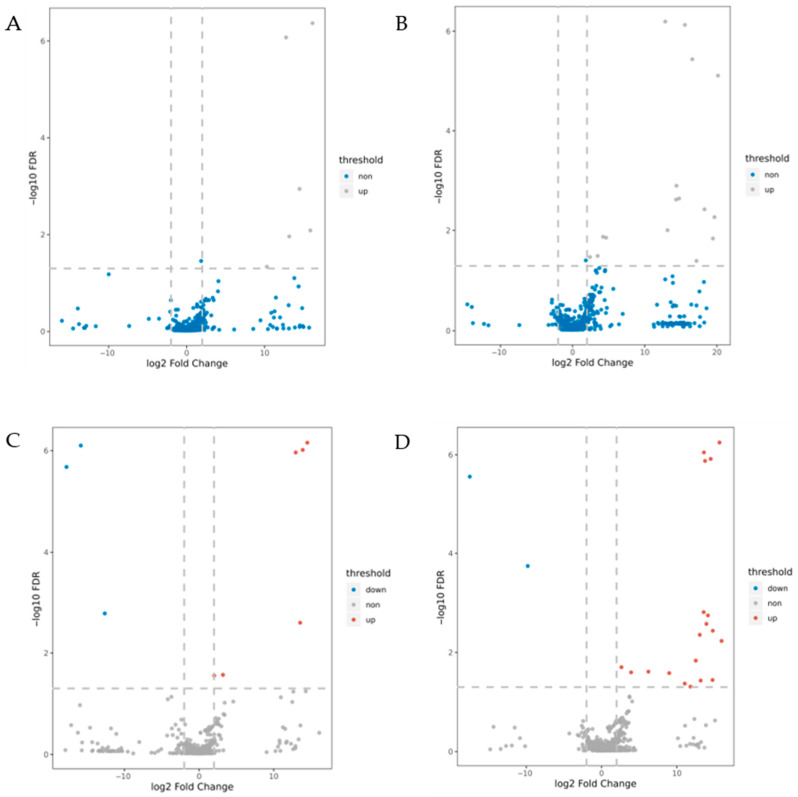
Volcano plots of DEPs. Note: (**A**–**D**): B24 vs. BCK, B48 vs. BCK, Q24 vs. QCK, Q48 vs. QCK; red: upregulated; blue: downregulated; gray: non-significant.

**Figure 5:**
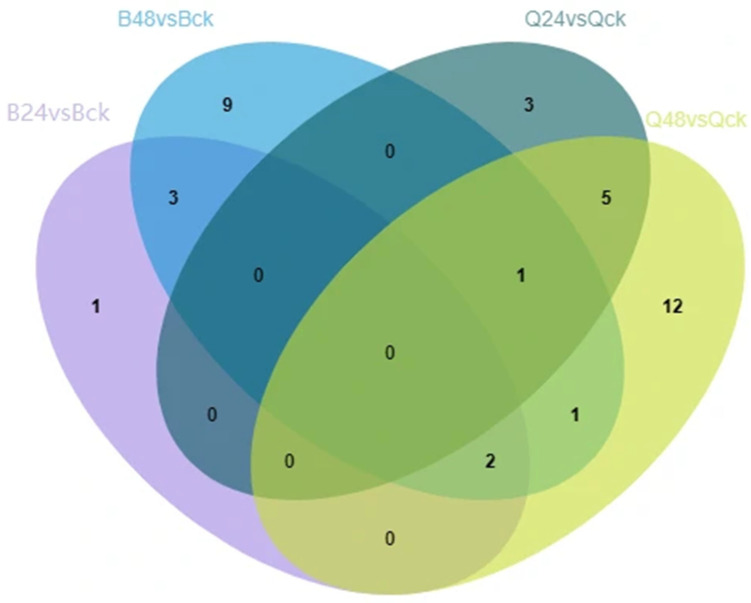
Venn diagram of DEPs between groups. Note: Non-overlapping regions indicate group-specific DEPs; overlapping regions denote shared DEPs.

This study employed data-independent acquisition (DIA) coupled with LC-MS/MS technology to systematically identify and quantify DEPs between B144 and Q319 under three experimental conditions: control (CK), 24 h low-temperature stress, and 48 h low-temperature stress. DEPs were defined as proteins exhibiting a fold change >2 or <0.5 (adjusted ANOVA *p* < 0.05, *FDR* < 0.05). Analysis of four comparison groups (B24 vs. BCK, B48 vs. BCK, Q24 vs. QCK, Q48 vs. QCK) revealed distinct DEP profiles. Notably, B144 showed exclusive upregulation of DEPs under stress: 6 DEPs in B24 vs. BCK and 16 DEPs in B48 vs. BCK. In contrast, the Q319 exhibited mixed regulation, with 9 DEPs (6 upregulated, 3 downregulated) in Q24 vs. QCK and 21 DEPs (19 upregulated, 2 downregulated) in Q48 vs. QCK. Volcano plots (Figure 4) visualized these DEPs, with red and blue dots representing upregulated and downregulated proteins, respectively, while gray dots indicated non-significant proteins. The dispersion coordinates (log2 fold change vs. −log10 *p*-value) demonstrated pronounced differential expression in B48 vs. BCK, reflecting extensive molecular reprogramming under prolonged stress. Venn diagram analysis (Figure 5) revealed no shared DEPs between B24 vs. BCK and Q24 vs. QCK, indicating divergent short-term stress responses. Four overlapping DEPs between B48 vs. BCK and Q48 vs. QCK suggested conserved pathways in prolonged stress adaptation. These results comprehensively delineate dynamic proteomic landscapes underlying low-temperature tolerance and sensitivity in maize.

### 3.5. GO Annotation and Enrichment Analysis

**Figure 6:**
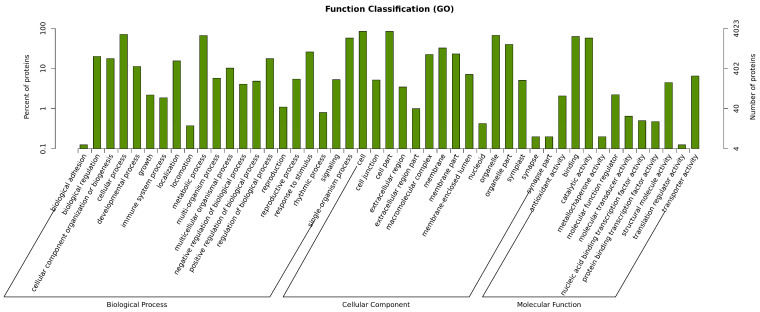
GO annotation. Note: the x-axis represents secondary-level GO terms, while the y-axis indicates the number of proteins annotated to each term (right side) and their percentage relative to the total protein count (left side). The bottom of the figure displays primary GO categories (from left to right): biological process, cellular component, and molecular function.

GO annotation analysis was performed on all identified proteins across three categories: biological process (BP), cellular component (CC), and molecular function (MF). As shown in Figure 6, among the 4288 identified proteins, 1962 proteins (annotation rate: ~45.7%) were annotated with GO terms. A total of 47 GO terms were significantly enriched, including 21 terms in BP, 15 in CC, and 11 in MF. Biological processes were primarily associated with cellular organization or biogenesis, biological regulation, developmental processes, cellular processes, immune system processes, growth and reproduction, localization, metabolic processes, multi-organism processes, multicellular organismal processes, regulation of biological processes, response to stimuli, rhythmic processes, single-organism processes, and signal transduction. Cellular components were enriched in terms such as cell junction, cell part, extracellular region, macromolecular complex, membrane part, membrane-enclosed lumen, organelle part, and synapse part. Molecular functions included molecular transducer activity, catalytic activity, nucleic acid-binding transcription factor activity, protein-binding transcription factor activity, transporter activity, and molecular function regulator.

**Figure 7:**
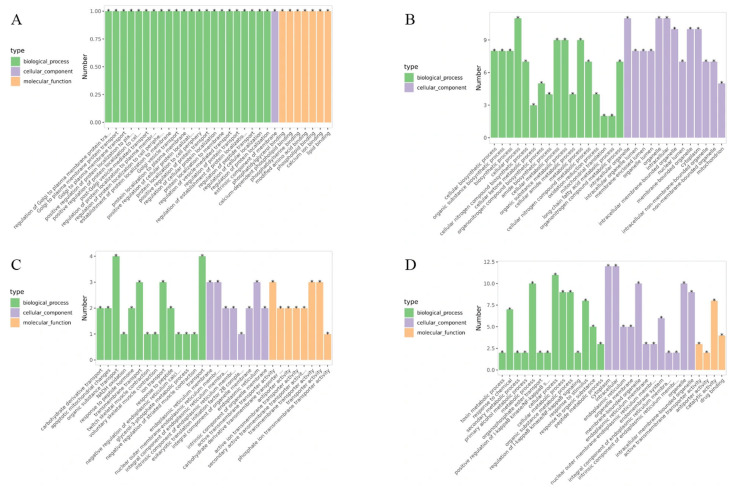
GO enrichment analysis of DEPs in maize under low-temperature stress. Note: (**A**) represents the enrichment of DEPs in leaves of B24 vs. BCK; (**B**) represents the enrichment of DEPs in leaves of B48 vs. BCK; (**C**) represents the enrichment of DEPs in leaves of Q24 vs. QCK; (**D**) represents the enrichment of DEPs in leaves of Q48 vs. QCK.

Differential protein expression analysis between B144 and Q319 was conducted to investigate their molecular mechanisms in low-temperature stress responses. GO functional annotation of the DEPs was performed. In the B24 vs. BCK group, DEPs were enriched in 46 biological processes, 18 cellular components, and 12 molecular functions. The top 30 pathways most significantly associated with low-temperature stress responses were identified based on GO hierarchical relationships and enrichment levels (Figure 7A). Notably, a shared gene (Gene ID: *A0A096QXB7*), annotated as a calcium-dependent phospholipid-binding protein belonging to the calcium-dependent membrane-associated protein family, was enriched across all three GO categories (Table 2). The enrichment of *A0A096QXB7* across all categories suggests its multifunctional role in coordinating calcium-mediated signaling and membrane stabilization, potentially mitigating low-temperature-induced membrane rigidification or facilitating stress signal transduction. These findings collectively demonstrate the integration of calcium signaling, metabolic reprogramming, and structural maintenance in maize low-temperature tolerance, with *A0A096QXB7* emerging as a pivotal candidate gene for cross-compartmental stress adaptation mechanisms.

In the B48 vs. BCK group, DEPs were associated with 777 biological processes, 112 cellular components, and 116 molecular functions. The top 30 pathways most significantly involved in low-temperature stress responses were identified (Figure 7B). Table 3 demonstrates that within the biological process category, differentially expressed genes were highly enriched in cellular biosynthesis, organic substance biosynthesis, nitrogen-containing compound metabolism, and cellular metabolic processes, highlighting the central role of biosynthetic and nitrogen-related pathways in cold adaptation. In the cellular component category, significant enrichment was observed for genes localized to cellular organelles, emphasizing the importance of organelle-specific functions in stress responses. Notably, no significant enrichment of differentially expressed genes was detected in the molecular function category, suggesting that molecular functional diversity may not be a primary driver of cold stress adaptation in this comparison group. These findings underscore the prioritization of biosynthetic and metabolic reprogramming over molecular functional specialization in maize under prolonged low-temperature stress, with cellular organelle dynamics emerging as a critical component of the adaptive machinery.

In the Q24 vs. QCK group, DEPs were enriched in 338 biological processes, 44 cellular components, and 77 molecular functions. The top 30 pathways predominantly involved in low-temperature stress responses were identified (Figure 7C). Table 4 revealed that within the biological process category, differentially expressed genes were strikingly enriched in carbohydrate derivative transport, with four genes enriched in organic substance transport, highlighting the critical role of transmembrane transport systems in maintaining metabolic homeostasis under low-temperature stress. Cellular component analysis showed significant enrichment of genes localized to the endoplasmic reticulum (ER) membrane and intrinsic components of cellular organelles, suggesting ER-mediated protein processing and organelle-specific adaptations as key mechanisms. Molecular function analysis identified three highly enriched GO terms: active transmembrane transporter activity, carbohydrate derivative transmembrane transporter activity, and antiporter activity, emphasizing the importance of ion and solute transport across membranes in cold adaptation. These findings underscore the centrality of transmembrane transport machinery, particularly carbohydrate and organic compound shuttling, in mediating maize responses to low-temperature stress. The enrichment of ER-associated components further implicates protein folding, modification, and secretion pathways in stress resilience, while antiporter activity may regulate ion balance to counteract cold-induced cellular dysregulation. Collectively, this analysis reveals that low-temperature-sensitive maize lines prioritize transport-driven metabolic adjustments and organelle-level adaptations to mitigate low-temperature damage.

In the Q48 vs. QCK group, DEPs were enriched in 570 biological processes, 67 cellular components, and 129 molecular functions. The top 30 pathways predominantly involved in low-temperature stress responses were identified (Figure 7D). Table 5 revealed significant enrichment in biological processes such as toxin metabolism, cellular metabolism, and organic substance metabolism, highlighting the importance of detoxification and metabolic adjustments under prolonged low-temperature stress. Cellular component analysis showed prominent enrichment in cytoplasmic regions, intracellular compartments, and organelle membranes, suggesting coordinated adaptations in subcellular organization and membrane-associated processes. Molecular function analysis identified active transmembrane transporter activity and antiporter activity as the most significantly enriched terms, with catalytic activity representing the largest proportion of enriched GO terms. These findings emphasize the centrality of transmembrane transport systems and enzymatic catalysis in cold adaptation, likely facilitating ion homeostasis, metabolite shuttling, and detoxification. The enrichment of organelle membrane-associated components further implicates membrane integrity maintenance and compartmentalized metabolic regulation in stress resilience. Collectively, this analysis underscores the interplay of detoxification, metabolic flexibility, and transport-driven cellular adjustments in maize responses to prolonged low-temperature stress, with catalytic and antiporter activities serving as key functional drivers to mitigate cold-induced metabolic dysregulation.

### 3.6. KEGG Pathway Analysis

**Figure 8:**
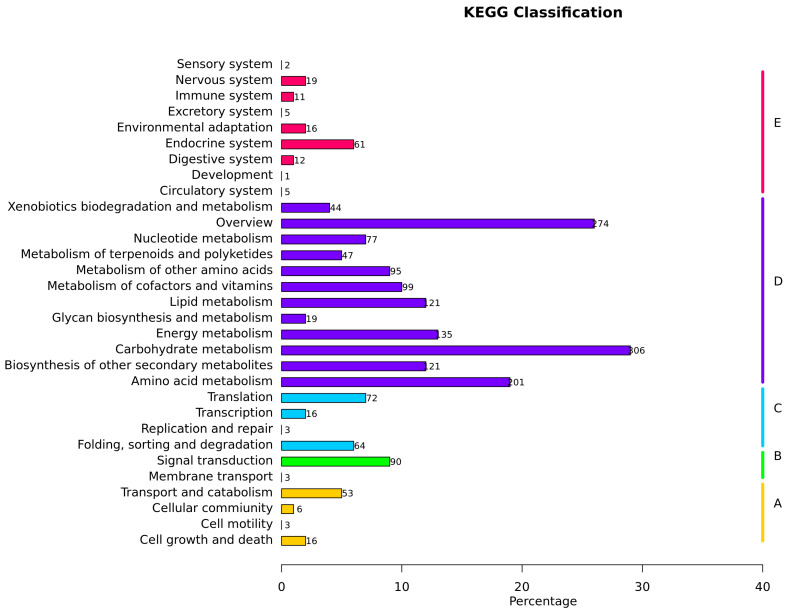
Enrichment analysis of KEGG metabolic pathways of proteins in maize responding to low-temperature stress. Note: the left and right sides of the vertical axis, respectively, represent the secondary and primary classification names of KEGG metabolic pathways. The horizontal axis represents the percentage of proteins annotated to the metabolic pathways in the total number of quantified proteins. The Arabic numerals on the right side of each horizontal bar in the figure indicate the number of proteins annotated to that pathway.

During low-temperature stress, protein functions are executed through coordinated actions of multiple proteins, and pathway analysis provides a comprehensive understanding of their biological processes and stress response mechanisms. To elucidate metabolic pathway alterations in maize leaves under low-temperature stress, identified proteins were mapped to KEGG pathways for annotation and enrichment analysis. A total of 31 pathways were significantly enriched (Figure 8), with prominent enrichment observed in carbohydrate metabolism (including amino sugar/nucleotide sugar metabolism, glycolysis/gluconeogenesis, pyruvate metabolism, and starch/sucrose metabolism), biosynthesis of other secondary metabolites (phenylpropanoid biosynthesis), nucleotide metabolism (purine metabolism), carbon metabolism, and amino acid biosynthesis. Notably, the highest protein distributions were detected in carbohydrate metabolism, carbon metabolism, and amino acid biosynthesis pathways, suggesting their critical roles in mediating maize responses to low-temperature stress. These findings highlight the centrality of energy metabolism, secondary metabolite production, and nitrogen assimilation in cold adaptation, with carbohydrate and amino acid pathways serving as key hubs for maintaining metabolic homeostasis under adverse conditions.

### 3.7. WGCNA

#### 3.7.1. Module Partitioning and Specific Module Identification

**Figure 9:**
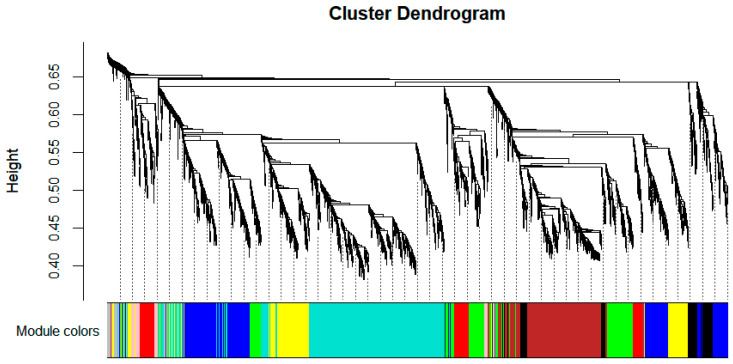
Hierarchical clustering heatmap of proteins. Note: the upper part is the hierarchical clustering dendrogram of proteins, and the lower part is the protein modules. Proteins that are closer in distance are classified into the same module.

**Figure 10:**
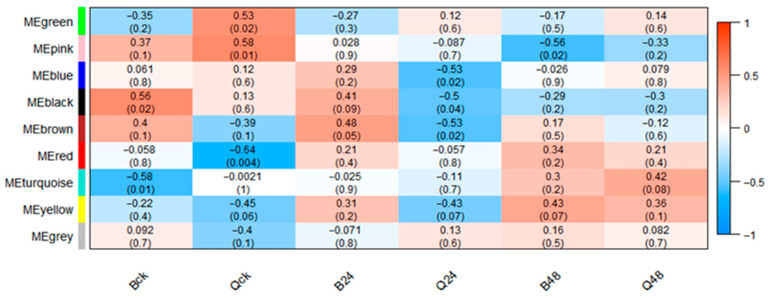
Correlation diagram between protein modules and sample groupings. Note: the color blocks on the far left represent the modules, and the color bar on the far right represents the range of correlations. In the heatmap in the middle part, the darker the color, the higher the correlation. Red indicates a positive correlation, and blue indicates a negative correlation. The number in each cell represents the correlation and significance.

To elucidate protein dynamics across treatments and explore maize responses to low-temperature stress, a weighted gene co-expression network was constructed using 4367 identified proteins. As shown in Figure 9, dynamic tree-cutting partitioned proteins with similar expression patterns into co-expression modules, yielding nine distinct modules: blue (756 proteins), green (539), black (232), brown (699), yellow (546), red (271), turquoise (1142), pink (110), and grey (72). These modules were visualized as branches and color-coded clusters in the hierarchical clustering dendrogram. Subsequently, module–trait association analysis was performed to identify representative modules for specific treatment groups (Figure 10). The brown module exhibited a significant positive correlation with B24 (*r* = 0.48, *p* < 0.05), while the blue module showed a strong negative correlation with Q24 (*r* = −0.53, *p* < 0.05). The pink module displayed the highest negative correlation with B48 (*r* = −0.56, *p* < 0.05), and the turquoise module was most associated with Q48 (*r* = 0.42), though this correlation lacked statistical significance (*p* > 0.05). These results highlight genotype- and duration-specific modules, with the brown and pink modules representing low-temperature-tolerant adaptation mechanisms in B144 and the blue module reflecting acute stress responses in Q319. The turquoise module’s weak association with Q48 suggests incomplete metabolic reprogramming in the low-temperature-sensitive line under prolonged stress.

#### 3.7.2. Functional Enrichment Analysis of Representative Modules

**Figure 11:**
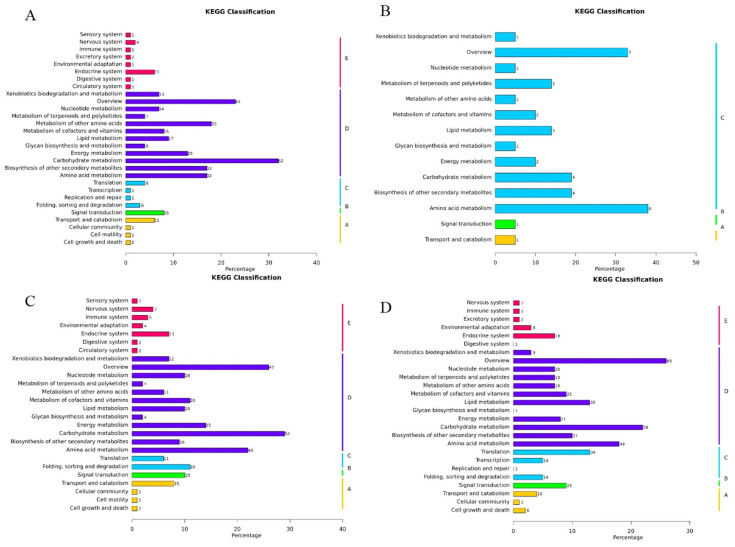
Analysis of the functions of representative modules. Note: (**A**) is the KEGG enrichment analysis of proteins in the representative module of B24; (**B**) is the KEGG enrichment analysis of proteins in the representative module of B48; (**C**) is the KEGG enrichment analysis of proteins in the representative module of Q24; (**D**) is the KEGG enrichment analysis of proteins in the representative module of Q48. On the left and right sides of the vertical axis, the secondary and primary classification names of the KEGG metabolic pathways, respectively, are shown. The horizontal axis represents the percentage of proteins annotated to the metabolic pathways in the total number of quantified proteins. The Arabic numerals on the right side of each horizontal bar in the figure indicate the number of proteins annotated to that pathway.

KEGG functional enrichment analysis of representative modules revealed distinct metabolic reprogramming strategies between B144 and Q319 under low-temperature stress. In B144, the brown module (24 h, 699 proteins) showed significant enrichment in phenylpropanoid biosynthesis, starch/sucrose metabolism, glutathione metabolism, 2-oxocarboxylic acid metabolism, and amino acid biosynthesis, reflecting early activation of antioxidant systems (e.g., glutathione), energy mobilization, and secondary metabolite production (Figure 11A). Prolonged low-temperature exposure (48 h, pink module with 110 proteins) shifted toward amino acid biosynthesis, alanine/aspartate/glutamate metabolism, carbon metabolism, and glycerolipid metabolism, emphasizing nitrogen assimilation and membrane stability (Figure 11B). In contrast, Q319 exhibited contrasting patterns: its blue module (24 h, 756 proteins) prioritized amino acid biosynthesis, carbon metabolism, glycolysis/gluconeogenesis, and pyruvate metabolism, indicative of acute reliance on core energy pathways (Figure 11C). Extended stress (48 h, turquoise module with 1142 proteins) further engaged fatty acid biosynthesis, pyrimidine metabolism, and sustained glycolysis/gluconeogenesis, suggesting compensatory nucleotide synthesis and persistent energy demands (Figure 11D). Notably, B144 demonstrated specialized stress adaptation through phased metabolic adjustments—transitioning from redox homeostasis to nitrogen/lipid metabolism—while Q319 maintained generalized metabolic upkeep with escalating fatty acid and nucleotide synthesis, highlighting genotype-specific survival mechanisms. These findings underscore that low-temperature tolerance in maize involves targeted activation of stress-responsive pathways, whereas sensitivity correlates with inefficient metabolic flexibility and prolonged energy-intensive processes.

#### 3.7.3. Hub Protein Analysis of Representative Modules

**Figure 12:**
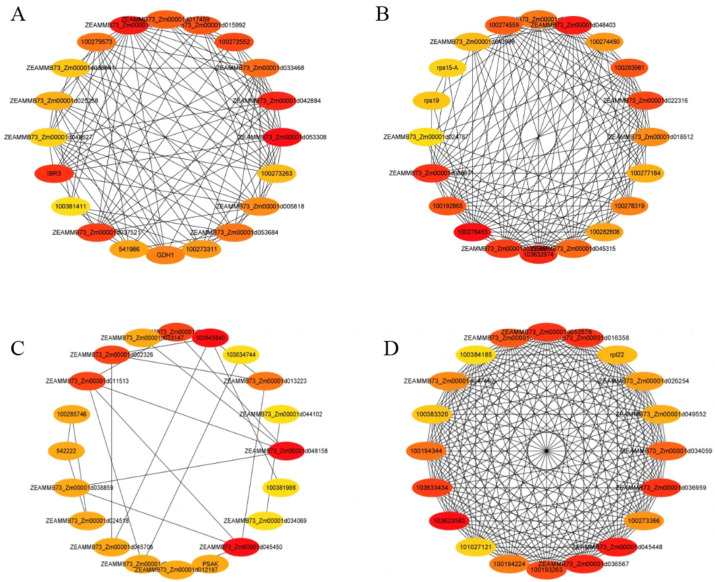
Analysis of hub proteins. Note: (**A**) represents the interaction network of hub proteins in the representative brown module of B24; (**B**) represents the interaction network of hub proteins in the representative blue module of Q24; (**C**) represents the interaction network of hub proteins in the representative pink module of B48; (**D**) represents the interaction network of hub proteins in the representative turquoise module of Q48.

Protein–protein interaction (PPI) networks for distinct modules were analyzed using the STRING database (https://www.string-db.org/) and visualized with Cytoscape 3.9.1 to identify hub proteins, defined as the most highly interconnected nodes within each module. As shown in Figure 12A, the top five hub proteins in the brown module (representing B144 under 24 h low-temperature stress) were identified as Zm00001d053308, Zm00001d016659, Zm00001d042884, IBR3, and Zm00001d037521, corresponding to an uncharacterized protein, acetyl-CoA synthetase, acyl-CoA oxidase, acyl-CoA dehydrogenase-like protein, and acyl-CoA oxidase 4, respectively. These findings highlight the central role of acetyl-CoA metabolism and fatty acid β-oxidation in early cold adaptation. Acetyl-CoA synthetase catalyzes the production of acetyl-CoA, a critical substrate for energy generation via the TCA cycle, while acyl-CoA oxidases drive fatty acid degradation into acetyl-CoA, providing both energy and metabolic intermediates. The co-occurrence of multiple acyl-CoA oxidases (e.g., acyl-CoA oxidase 4) further underscores the regulatory importance of lipid metabolism in maintaining cellular homeostasis under low-temperature stress. This module-specific hub network suggests that cold-tolerant maize lines prioritize rapid metabolic reprogramming to sustain energy supply and redox balance during initial cold exposure.

As shown in Figure 12B, the top five hub proteins in the blue module (representing the Q319 under 24 h low-temperature stress) were identified as LOC100276453, Zm00001d048403, LOC103632974, Zm00001d038871, and Zm00001d033596, corresponding to 26S proteasome non-ATPase regulatory subunit 7 homolog A, proteasome subunit alpha type, proteasome subunit beta, Mov34/MPN/PAD-1 family protein, and proteasome subunit beta, respectively. This module is dominated by proteasome-associated proteins, emphasizing the critical role of proteasome-mediated protein turnover in cold-sensitive responses. The 26S proteasome regulatory subunit homolog and alpha/beta subunits are essential for proteolytic activity, targeting misfolded or damaged proteins for degradation under stress conditions. The Mov34/MPN/PAD-1 family protein, a conserved component of the proteasome lid subcomplex, further supports substrate recognition and processing. The prominence of multiple proteasome subunits highlights the reliance of cold-sensitive lines on protein homeostasis maintenance through accelerated degradation of stress-damaged proteins. This contrasts with the metabolic adaptation strategies observed in cold-tolerant lines, suggesting that proteostatic imbalance may contribute to reduced cold tolerance in Q319 during early stress exposure.

As shown in Figure 12C, the top five hub proteins in the pink module (representing B144 under 48 h low-temperature stress) were identified as LOC103645840, Zm00001d045450, Zm00001d048158, Zm00001d037873, and Zm00001d002326, corresponding to D-3-phosphoglycerate dehydrogenase, 3-phosphoshikimate 1-carboxyvinyltransferase, small ribosomal subunit protein eS1, elongation factor 1-α, and adenosylmethionine aminotransferase 1, respectively. This module reflects a coordinated regulatory network involving serine biosynthesis, translation elongation, and methyl group metabolism during prolonged cold exposure. D-3-phosphoglycerate dehydrogenase catalyzes the rate-limiting step in serine biosynthesis, providing precursors for glutathione production to counteract oxidative stress. 3-phosphoshikimate 1-carboxyvinyltransferase, a key enzyme in the shikimate pathway, supports aromatic amino acid synthesis, which is critical for secondary metabolite production under stress. Small ribosomal subunit protein eS1 and elongation factor 1-α jointly enhance translational efficiency, facilitating the synthesis of cold-responsive proteins such as osmoprotectants and antioxidants. Adenosylmethionine aminotransferase 1 modulates methyl group metabolism, influencing S-adenosylmethionine (SAM) availability for methylation reactions essential to epigenetic and metabolic regulation. These findings highlight B144’s adaptive strategy under sustained cold stress, integrating antioxidant defense, protein synthesis, and metabolic flexibility to maintain cellular homeostasis. The prominence of ribosomal and translational regulators underscores the importance of efficient protein production in long-term cold tolerance.

As shown in Figure 12D, the top five hub proteins in the turquoise module (representing the Q319 under 48h low-temperature stress) were identified as LOC103633583, Zm00001d045448, Zm00001d036567, Zm00001d036959, and Zm00001d016358, corresponding to 40S ribosomal protein S20-1, 40S ribosomal protein S20-1, 40S ribosomal protein S14-3, elongation factor gamma1, and putative elongation factor 1-gamma 2, respectively. This module is characterized by a strong enrichment of ribosomal proteins and translational elongation factors, reflecting the sustained demand for ribosome biogenesis and protein synthesis in cold-stressed plants. The dual occurrence of 40S ribosomal protein S20-1 (LOC103633583 and Zm00001d045448) underscores its critical role in maintaining ribosomal stability and translational fidelity under prolonged low-temperature conditions. 40S ribosomal protein S14-3 (Zm00001d036567) further contributes to small subunit assembly and mRNA decoding. The presence of elongation factor gamma1 (Zm00001d036959) and putative elongation factor 1-gamma 2 (Zm00001d016358) highlights the importance of translational elongation in synthesizing stress-responsive proteins such as molecular chaperones and cold-induced enzymes. These findings suggest that cold-sensitive maize lines like Q319 prioritize ribosome-mediated translational machinery during extended cold stress, potentially compensating for impaired metabolic adaptation mechanisms observed in tolerant genotypes. However, the persistent reliance on ribosomal activity may reflect inefficiencies in redirecting energy resources toward stress-specific pathways, contributing to reduced cold tolerance in sensitive lines. This contrasts with the metabolic flexibility and antioxidant-focused strategies seen in low-temperature-tolerant B144, emphasizing genotype-specific survival mechanisms under prolonged low-temperature stress.

### 3.8. Hub Gene Validation by qPCR

**Figure 13:**
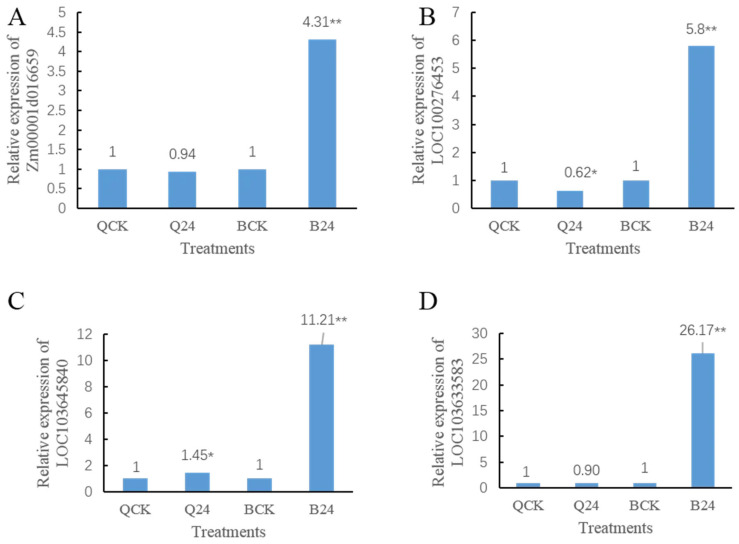
Detection of hub genes by qPCR. Note: (**A**) represents the expression level of *Zm00001d016659*; (**B**) represents the expression level of *LOC100276453*; (**C**) represents the expression level of *LOC103645840*; (**D**) represents the expression level of *LOC103633583*. ** denote levels of significance at *p* < 0.001, * denote levels of significance at *p* < 0.05.

We validated the expression of four hub genes of the brown module, blue module, pink module, and turquoise module. The maize *Actin1* gene was used as an internal control to standardize the data. qPCR results showed that the expression of all the tested genes was consistent with the proteomic data (Figure 13), confirming their roles as key regulators in maize’s low-temperature stress response.

## 4. Discussion

This study investigated differential protein expression patterns in maize inbred lines with contrasting low-temperature tolerance using DIA-based proteomic quantification. The results revealed significant differences in the number of DEPs between the low-temperature-tolerant line B144 and the low-temperature-sensitive line Q319 under low-temperature treatment, with an increase in DEP numbers at 48 h compared to 24 h. Four commonly upregulated DEPs—including ribosomal proteins (A0A3L6DTV4 and B6STW7); **oxidase protein (A0A1D6KR63);** glycerol-3-phosphate permease (B6ST65); and actin (A0A1D6NCZ8)—were identified, which are potentially crucial for maize’s low-temperature tolerance mechanism. These proteins are proposed as potential key targets in maize low-temperature tolerance mechanisms based on their established roles in plant low-temperature stress responses. Ribosomal proteins (RPs), as core ribosomal components, not only participate in protein translation but also regulate low-temperature stress adaptation. Transcriptomic investigations have shown that in cold-tolerant maize lines, cold stress leads to the upregulation of ribosomal pathway genes such as RPS6 and RPL23 [28]. KEGG enrichment analysis further validates the involvement of these genes in the cold response, highlighting their significance in the molecular mechanisms of cold tolerance. Research on Arabidopsis has revealed that the ribosomal processing protein STCH4/REIL2 plays a crucial role in regulating the translation efficiency of the CBF transcription factor under cold stress. The knockout mutant of STCH4 (stch4) exhibits a decreased cold tolerance, indicating the importance of STCH4/REIL2 in cold adaptation processes [29]. Through proteomic and genetic analyses in Arabidopsis, it has been suggested that the status of ribosomes may modulate cold signaling via feedback regulation mechanisms; however, whether this mechanism operates in maize remains to be confirmed. This finding implies a complex interplay between ribosomal function and cold stress responses at the molecular level in Arabidopsis, which may not directly apply to maize. Liu et al. identified that under cold stress, Arabidopsis HsfA1d activates ribosomal protein genes, including RPL18 and RPS5, by binding to their promoters [30]. This activation promotes hypocotyl elongation through a CBF-independent pathway, uncovering a novel regulatory mechanism in plant responses to cold stress. **The aforementioned oxidase protein (A0A1D6KR63) is a key enzyme in fatty acid β-oxidation, which initiates the breakdown of fatty acids into acetyl-CoA and other metabolic intermediates to supply energy and precursors for adaptive responses; it may also regulate jasmonate biosynthesis through fatty acid metabolism to participate in plant cold stress signaling—while ROS scavenging is mainly achieved by antioxidant enzymes such as peroxidases (e.g., PRX62 and PRX69) reported in Arabidopsis.** Oxidase proteins are essential components of antioxidant systems. They contribute significantly to maintaining cellular homeostasis by scavenging reactive oxygen species (ROS) and alleviating oxidative damage [31,32]. A GWAS analysis of 108 Arabidopsis accessions has identified peroxidases PRX62 and PRX69 as key regulators responsible for maintaining ROS homeostasis and cell wall stability during cold-induced root hair growth [33]. These peroxidases likely play a vital role in protecting plant cells from oxidative stress under cold conditions. Glycerol-3-phosphate permease, which is associated with phosphorus transport, may play a role in coordinating phosphorus metabolism adaptation during cold stress. Studies on Arabidopsis have shown that the AtG3PP1-AtG3PP5 genes exhibit differential expression under phosphorus-deficient conditions [34]. This differential expression suggests that these genes may contribute to environmental adaptation by regulating phosphorus metabolism, potentially having implications for plant responses to cold stress. Actin, a fundamental component of the cytoskeleton, not only maintains cellular integrity but also participates in cold signal transduction through interactions with signaling proteins [35,36]. Additionally, actin contributes to antioxidant defenses, highlighting its multifunctional role in plant responses to cold stress. Its involvement in both structural and signaling aspects of the cell under cold conditions further emphasizes the complexity of the cold tolerance mechanisms of plants.

Numerous studies have established a strong connection between plant responses to low-temperature stress and carbohydrate and amino acid metabolism. This relationship holds significant implications for various aspects of plant science, particularly in the context of improving crop performance under cold stress conditions. Carbohydrates contribute to multilayered cold tolerance mechanisms through dynamic accumulation, regulation of metabolic enzyme activities, and coordinated gene network interactions under cold stress [37,38]. For example, Shahryar et al. [39] conducted research on wheat genotypes with varying cold tolerance levels. They found that the cold-tolerant cultivars Norstar and Gerdish, when subjected to a −5 °C treatment for 24 h, had lower electrolyte leakage indices and a notable increase in the accumulation of sucrose, glucose, and fructose. Concurrently, transcriptional alterations were detected in genes encoding carbohydrate-metabolizing enzymes. This clearly underscores the crucial role of carbohydrate metabolism in plant adaptation to cold stress. Orzechowski et al. analyzed the carbohydrate dynamics in potato leaves under cold stress. Their findings indicated that short-term chilling led to transient starch accumulation, followed by a decline. Moreover, at the end of light periods, there was a significant rise in glucose and fructose levels. These changes were correlated with the upregulated transcription of genes responsible for initiating starch degradation, enhanced acid invertase activity, and the activation of glucan phosphorylase [40]. Zeng’s study elucidated that calcium-dependent protein kinase CPK17 regulates COOL1 protein stability via phosphorylation, establishing an interconnected regulatory network between cold signaling and sugar metabolism [41]. In maize, ZmCPK21 (ortholog of Arabidopsis CPK17) also phosphorylates ZmCOOL1 but with a lower phosphorylation efficiency, which may explain why maize requires higher COOL1 expression levels under cold stress compared to Arabidopsis—again reflecting interspecies differences in signaling pathway kinetics that should be considered when extrapolating findings across species. Amino acid biosynthesis pathways are similarly modulated during cold adaptation. Yang’s integrated transcriptomic–metabolomic analysis of cold-tolerant and cold-sensitive common bean varieties identified key regulatory networks involving amino acid biosynthesis, with enhanced expression of amino acid-related genes in both genotypes contributing to cold adaptation [42]. Cold stress has a profound impact on the expression of genes involved in amino acid biosynthesis, which in turn affects protein synthesis and stability. Jiang et al. utilized high-throughput LC-MS/MS to profile metabolites in cold-stressed maize leaves. Their study revealed that the transcription factor *ZmICE1* modulates cold tolerance by regulating the tyrosine/arginine/proline metabolism pathways. *ZmICE1* binds directly to the promoters of genes encoding rate-limiting enzymes and transporters in amino acid metabolism, thus playing a crucial role in maintaining metabolic homeostasis [43]. Yuan’s proteomic investigation of *Brassica campestris* demonstrated enhanced carbohydrate and amino acid metabolism as central strategies for cold acclimation [44]. Cheng’s combined metabolomic–transcriptomic analysis of tea cultivars further revealed differential accumulation of amino acid biosynthesis-related genes/metabolites between cold-tolerant and cold-sensitive varieties, suggesting their contribution to genotype-dependent cold tolerance [45]. Our findings align with these established mechanisms, showing significant enrichment of differentially expressed proteins in carbohydrate and amino acid metabolic pathways in maize leaves under cold stress. These results were corroborated by KEGG functional enrichment analysis of WGCNA modules, confirming these pathways as critical biological processes responsive to low-temperature stress. **Collectively, the comparison across maize, wheat, rice, cotton, and Arabidopsis highlights that while core cold tolerance mechanisms (e.g., RP-mediated translation, ROS scavenging, carbohydrate/amino acid metabolism) are conserved, the specific genes, expression kinetics, and metabolic fluxes exhibit species-specific adaptations—underscoring the importance of contextualizing maize findings within a crop-specific framework rather than overgeneralizing from model plants or other crops.**

In this study, we employed WGCNA to identify co-expressed protein modules under different treatment conditions and to pinpoint hub proteins within these networks. Proteomic profiling of the low-temperature-tolerant inbred line B144 and low-temperature-sensitive line Q319 revealed distinct response patterns to low-temperature stress. Under 24 h low-temperature stress, hub proteins in B144 were predominantly associated with acetyl-CoA synthetase and acyl-CoA oxidase, while Q319 exhibited hub proteins linked to the proteasome. Following 48 h low-temperature stress, B144 showed enrichment of hub proteins, including D-3-phosphoglycerate dehydrogenase, ribosomal small subunit protein eS1, elongation factor 1-α, and adenosylmethionine aminotransferase 1, whereas Q319 displayed hub proteins primarily related to ribosomal proteins and elongation factors. Studies have demonstrated that acetyl-CoA synthetase generates acetyl-CoA, a critical substrate for the tricarboxylic acid (TCA) cycle, which provides energy to sustain normal physiological functions in plants under low-temperature stress [46]. Acyl-CoA oxidase, a key enzyme in fatty acid β-oxidation, initiates the breakdown of fatty acids into acetyl-CoA and other metabolic intermediates, supplying energy and precursors for adaptive responses. Furthermore, acyl-CoA oxidase may regulate jasmonate biosynthesis through fatty acid metabolism, thereby participating in plant cold stress signaling and modulating cold tolerance [47]. The proteasome influences plant cold adaptation by regulating the expression of rate-limiting enzymes in amino acid metabolism pathways, which in turn affects amino acid synthesis and turnover [48]. Under cold stress, the accumulation of reactive oxygen species (ROS) in plant cells triggers the upregulation of D-3-phosphoglycerate dehydrogenase, which catalyzes serine synthesis. Serine serves as a precursor for glutathione biosynthesis, enhancing antioxidant capacity to scavenge excess ROS and alleviate oxidative damage [49]. Studies have reported that ribosomal proteins are associated with negative regulation of cold stress responses. Specifically, the *AtPRMT3-RPS2B* complex acts as a negative regulator of freezing tolerance in plants. *AtPRMT3* cooperates with the ribosomal small subunit protein RPS2B to coordinate nuclear processing of ribosomal precursors. Loss-of-function mutations in the *AtPRMT3-RPS2B* complex enhance the translation of stress-responsive genes while suppressing the translation of growth- and development-related genes, ultimately leading to a cold-tolerant phenotype in plants [50]. In maize, *ZmPRMT3* (ortholog of *AtPRMT3*) interacts with *ZmRPS2B*, but the complex has a lower affinity for ribosomal precursors, which may explain why maize requires higher *ZmPRMT3* expression to achieve the same regulatory effect as Arabidopsis—this represents an example of interspecies differences in protein–protein interaction kinetics that suggest caution when extrapolating findings from Arabidopsis to maize. Additionally, cold stress induces the upregulation of elongation factor 1-α (EF1-α), which facilitates the synthesis of cold-responsive proteins involved in osmoregulation and antioxidant defense. These proteins collectively maintain cellular osmotic balance and mitigate oxidative stress, thereby improving plant survival under low-temperature conditions [51]. Together, these findings highlight the coordinated roles of metabolic enzymes, ribosomal machinery, and translational regulators in orchestrating plant adaptation to cold stress through energy provision, redox homeostasis, and stress-responsive protein synthesis.

## 5. Conclusions

In the context of ongoing global climate change, temperature fluctuations are becoming increasingly unpredictable. This has posed a significant threat to maize production in numerous regions around the world, including North America, Europe, and certain parts of Africa and South America, where low-temperature stress has emerged as a major challenge. Through integrated proteomic and functional analyses, this study systematically elucidated the molecular regulatory mechanisms underlying the low-temperature stress responses in the low-temperature-tolerant maize inbred line B144 and the low-temperature-sensitive inbred line Q319. We preliminarily identified low-temperature responsive genes and proteins in maize seedlings. These identified elements offer potential targets for the development of low-temperature-resistant molecular markers and gene-editing- based breeding strategies on a global scale. The findings of this research lay a theoretical groundwork for screening low-temperature-tolerant germplasm resources and breeding maize varieties with enhanced low-temperature tolerance. Ultimately, these results contribute to the sustainable development of maize production in the face of changing climate conditions.

## Figures and Tables

**Table 2:** GO classification of differentially expressed genes in the B24 vs. BCK group.

Type	GO Annotation	GO_ID	*p*-Value	Count
Biological process	Regulation of Golgi to plasma membrane protein transport	GO:0042996	0.0003	1
	Golgi to plasma membrane protein transport	GO:0043001	0.0010	1
	Golgi to plasma membrane transport	GO:0006893	0.0015	1
	Positive regulation of protein localization to plasma membrane	GO:1903078	0.0016	1
	Positive regulation of protein localization to cell periphery	GO:1904377	0.0018	1
	Post-Golgi vesicle-mediated transport	GO:0006892	0.0024	1
	Regulation of protein localization to plasma membrane	GO:1903076	0.0028	1
	Regulation of protein localization to cell periphery	GO:1904375	0.0035	1
	Establishment of protein localization to membrane	GO:0090150	0.0057	1
	Golgi vesicle transport	GO:0048193	0.0064	1
	Protein localization to plasma membrane	GO:0072659	0.0072	1
	Positive regulation of cellular protein localization	GO:1903829	0.0076	1
	Protein localization to cell periphery	GO:1990778	0.0088	1
	Positive regulation of protein transport	GO:0051222	0.0112	1
	Regulation of cellular protein localization	GO:1903827	0.0138	1
	Protein localization to membrane	GO:0072657	0.0138	1
	Regulation of vesicle-mediated transport	GO:0060627	0.0150	1
	Regulation of protein transport	GO:0051223	0.0183	1
	Regulation of establishment of protein localization	GO:0070201	0.0191	1
	Regulation of peptide transport	GO:0090087	0.0194	1
	Regulation of cellular localization	GO:0060341	0.0207	1
	Regulation of protein localization	GO:0032880	0.0267	1
Cellular component	Extrinsic component of membrane	GO:0019898	0.0076	1
Molecular function	Phosphatidylglycerol binding	GO:1901611	0.0003	1
	Calcium-dependent phospholipid binding	GO:0005544	0.0014	1
	Phosphatidylserine binding	GO:0001786	0.0016	1
	Modified amino acid binding	GO:0072341	0.0027	1
	Phospholipid binding	GO:0005543	0.0105	1
	Calcium ion binding	GO:0005509	0.0154	1
	Lipid binding	GO:0008289	0.0183	1

**Table 3:** GO classification of enriched differentially expressed genes in the B48 vs. BCK group.

Type	GO Annotation	GO_ID	*p*-Value	Count
Biological process	Cellular biosynthetic process	GO:0044249	5.06 × 10^−6^	8
	Organic substance biosynthetic process	GO:1901576	5.83 × 10^−6^	8
	Biosynthetic process	GO:0009058	6.87 × 10^−6^	8
	Cellular process	GO:0009987	7.44 × 10^−6^	11
	Cellular nitrogen compound biosynthetic process	GO:0044271	1.70 × 10^−5^	7
	Cellular ketone metabolic process	GO:0042180	2.05 × 10^−5^	3
	Organonitrogen compound biosynthetic process	GO:1901566	2.87 × 10^−5^	5
	Amide biosynthetic process	GO:0043604	2.88 × 10^−5^	4
	Cellular metabolic process	GO:0044237	5.96 × 10^−5^	9
	Organic substance metabolic process	GO:0071704	7.78 × 10^−5^	9
	Cellular amide metabolic process	GO:0043603	9.74 × 10^−5^	4
	Metabolic process	GO:0008152	0.0001	9
	Cellular nitrogen compound metabolic process	GO:0034641	0.0001	7
	Oxidation–reduction process	GO:0055114	0.0001	4
	Mitochondrial translation	GO:0032543	0.0002	2
	Long-chain fatty acid metabolic process	GO:0001676	0.0002	2
	Organonitrogen compound metabolic process	GO:1901564	0.0003	7
Cellular component	Intracellular organelle	GO:0043229	9.69 × 10^−7^	11
	Intracellular organelle lumen	GO:0070013	1.19 × 10^−6^	8
	Membrane-enclosed lumen	GO:0031974	1.19 × 10^−6^	8
	Organelle lumen	GO:0043233	1.19 × 10^−6^	8
	Organelle	GO:0043226	1.32 × 10^−6^	11
	Intracellular	GO:0005622	3.99 × 10^−6^	11
	Intracellular membrane-bounded organelle	GO:0043231	6.54 × 10^−6^	10
	Nuclear lumen	GO:0031981	1.16 × 10^−5^	7
	Membrane-bounded organelle	GO:0043227	1.29 × 10^−5^	10
	Cytoplasm	GO:0005737	1.35 × 10^−5^	10
	Intracellular non-membrane-bounded organelle	GO:0043232	2.20 × 10^−5^	7
	Non-membrane-bounded organelle	GO:0043228	2.26 × 10^−5^	7
	Mitochondrion	GO:0005739	6.98 × 10^−5^	5

**Table 4:** GO classification of enriched differentially expressed genes in the Q24 vs. QCK group.

Type	GO Annotation	GO_ID	*p*-Value	Count
Biological process	Carbohydrate derivative transport	GO:1901264	5.88 × 10^−5^	2
	Apoptotic mitochondrial changes	GO:0008637	0.0001	2
	Organic substance transport	GO:0071702	0.0002	4
	NADH oxidation	GO:0006116	0.0010	1
	Response to peptide hormone	GO:0043434	0.0010	2
	Transmembrane transport	GO:0055085	0.0010	3
	Twitch skeletal muscle contraction	GO:0014721	0.0011	1
	Voluntary skeletal muscle contraction	GO:0003010	0.0011	1
	Ion transport	GO:0006811	0.0013	3
	Response to peptide	GO:1901652	0.0015	2
	Negative regulation of endoplasmic reticulum calcium ion concentration	GO:0032471	0.0015	1
	Glycerol-3-phosphate metabolic process	GO:0006072	0.0016	1
	Negative regulation of striated muscle contraction	GO:0045988	0.0016	1
	transport	GO:0006810	0.0018	4
Cellular component	Endoplasmic reticulum membrane	GO:0005789	3.79 × 10^−5^	3
	Nuclear outer membrane–endoplasmic reticulum membrane network	GO:0042175	4.25 × 10^−5^	3
	Integral component of endoplasmic reticulum membrane	GO:0030176	0.0002	2
	Intrinsic component of endoplasmic reticulum membrane	GO:0031227	0.0002	2
	Eukaryotic translation initiation factor 2B complex	GO:0005851	0.0010	1
	Integral component of organelle membrane	GO:0031301	0.0011	2
	Endoplasmic reticulum	GO:0005783	0.0013	3
	Intrinsic component of organelle membrane	GO:0031300	0.0014	2
Molecular function	Active transmembrane transporter activity	GO:0022804	1.26 × 10^−5^	3
	Carbohydrate derivative transmembrane transporter activity	GO:1901505	1.84 × 10^−5^	2
	Antiporter activity	GO:0015297	6.38 × 10^−5^	2
	Active ion transmembrane transporter activity	GO:0022853	0.0005	2
	Secondary active transmembrane transporter activity	GO:0015291	0.0005	2
	Transmembrane transporter activity	GO:0022857	0.0005	3
	Transporter activity	GO:0005215	0.0007	3
	Phosphate ion transmembrane transporter activity	GO:0015114	0.0015	1

**Table 5:** GO classification of enriched differentially expressed genes in the Q48 vs. QCK group.

Type	GO Annotation	GO_ID	*p*-Value	Count
Biological process	Toxin metabolic process	GO:0009404	3.4371 × 10^−5^	2
	Response to chemical	GO:0042221	0.0001	7
	Secondary metabolic process	GO:0019748	0.0002	2
	Primary alcohol metabolic process	GO:0034308	0.0002	2
	Metabolic process	GO:0008152	0.0003	10
	Organophosphate ester transport	GO:0015748	0.0007	2
	Positive regulation of I-kappaB kinase/NF-kappaB signaling	GO:0043123	0.0008	2
	Cellular process	GO:0009987	0.0009	11
	Cellular metabolic process	GO:0044237	0.0011	9
	Organic substance metabolic process	GO:0071704	0.0014	9
	Regulation of I-kappaB kinase/NF-kappaB signaling	GO:0043122	0.0016	2
	Response to stimulus	GO:0050896	0.0018	8
	Response to organic substance	GO:0010033	0.0019	5
	Peptide metabolic process	GO:0006518	0.0019	3
Cellular component	Cytoplasm	GO:0005737	5.83372 × 10^−6^	12
	Intracellular	GO:0005622	5.78814 × 10^−5^	12
	Endoplasmic reticulum	GO:0005783	0.0002	5
	Organelle membrane	GO:0031090	0.0005	5
	Membrane-bounded organelle	GO:0043227	0.0005	10
	Endoplasmic reticulum membrane	GO:0005789	0.0006	3
	Nuclear outer membrane–endoplasmic reticulum Membrane network	GO:0042175	0.0007	3
	Endomembrane system	GO:0012505	0.0011	6
	Integral component of endoplasmic reticulum membrane	GO:0030176	0.0012	2
	Intrinsic component of endoplasmic reticulum membrane	GO:0031227	0.0013	2
	Organelle	GO:0043226	0.0013	10
	Intracellular membrane-bounded organelle	GO:0043231	0.0018	9
Molecular function	Active transmembrane transporter activity	GO:0022804	0.0002	3
	Antiporter activity	GO:0015297	0.0004	2
	Catalytic activity	GO:0003824	0.0009	8
	Drug binding	GO:0008144	0.0021	4

## Data Availability

No new data were created or analyzed in this study.
